# Relationship between traditional Chinese medicine body constitution and sleep quality among high-speed railway crew in Beijing, China: A cross-sectional study

**DOI:** 10.1097/MD.0000000000044563

**Published:** 2025-09-12

**Authors:** Bozhen Huang, Meijiao Zhou, Shanshan Song, Min Jiang, Lei Liu, Liang Wang, Xinqian Liu, Jiaxuan Lyu, Guoyu Wang, Xinxin Liu, Fangzhou Jin

**Affiliations:** a Beijing Shijitan Hospital, Capital Medical University, Beijing, China; b First Teaching Hospital of Tianjin University of Traditional Chinese Medicine, Tianjin, China; c Beijing University of Chinese Medicine, Beijing, China.

**Keywords:** body constitution, constitution in Chinese medicine questionnaire, high-speed railway crew, sleep quality

## Abstract

This study aimed to investigate the relationship between traditional Chinese Medicine (TCM) body constitution and sleep quality among high-speed railway crew in Beijing, China. Evaluate TCM body constitution and sleep quality by using the constitution in Chinese medicine questionnaire (CCMQ) and the Pittsburgh sleep quality index (PSQI). From March 19, 2022, to November 20, 2023, a total of 799 questionnaires were distributed and returned 742 copies of the CCMQ and PSQI. The univariate analysis results showed significant association between sleep quality and Yang-deficiency constitution, Yin-deficiency constitution, phlegm-dampness constitution, dampness-heat constitution, blood-stasis constitution, Qi-stagnation constitution, Inherited-special constitution, academic degree (*P* < .05). In the multivariate analysis, Yin-deficiency constitution (OR = 2.492, 95% CI = 1.824–3.405) and Qi-stagnation constitution (OR = 2.097, 95% CI = 1.429–3.076) were associated with the sleep quality (*P* < .001). This cross-sectional study showed an association between Yin-deficiency and Qi-stagnation constitutions and sleep disorder in Beijing high-speed railway crew. However, the cross-sectional design precludes causal inference, and improving TCM body constitution may not necessarily lead to improved sleep quality. Further longitudinal research is needed to establish causal relationships. Nevertheless, this study provides a case for the potential role of TCM in supporting occupational health.

## 1. Introduction

The development of high-speed railway is a sign of the progress of public service in modern society and high-speed railway crew play an important role in safe transportation. As a service industry, the characteristics of high-speed railway crew’s work are full of noise,^[1]^ irregular shifts^[2]^ and high psychological pressure,^[3]^ which seriously affect the normal sleep circadian rhythm of high-speed railway crew. A good sleep is significant for high-speed railway crew. Adequate recovery process is needed by sleeping after intense work, a study showed that sleep <5 hours or awake for more than 16 hours in the day before work can significantly increases the possibility of fatigue-related damage and errors on the job.^[4]^ The Canadian Railway Accident Investigation Report found that bad sleep quality (BSQ) caused fatigue were clearly identified as the main reason of railway accidents.^[5]^ Therefore, the sleep quality of high-speed railway crew can directly affect whether the transportation work can be successfully completed.

Sleep disorder is mainly characterized by difficulty in falling asleep, short sleep time and waking up easily. Some studies proved that sleep disorder is a potential risk factor for cardiovascular diseases,^[6,7]^ metabolic diseases^[8]^ and death.^[9]^ Sleep disorder has become a public health problem in the world. There is an urgent need for effective prevent sleep disorder. However, some studies have found that there are individual differences in people’s tolerance to work environment.^[10,11]^ A cross-sectional study of Chinese railway workers suggested that environmental effects on sleep disorder may be dominated by genes.^[12]^ These individual differences are similar to the concept of “body constitution” in traditional Chinese Medicine (TCM). The “body constitution” refers to the relatively stable characteristic formed on the basis of heredity and acquired cultivation.^[13]^

There are some differences in adapting to the environment and the tendency of disease among TCM body constitution. It has been shown that there is a close connection between TCM body constitution and sleep quality.^[14]^ Therefore, TCM body constitution can predict who is prone to have sleep disorder. At present, there is a gap in the correlation analysis between TCM body constitution and sleep quality of high-speed railway crew. We hypothesize that TCM body constitution types may be associated with sleep quality among high-speed railway crew in Beijing, China. However, due to the cross-sectional nature of the study, we can not infer causality. Specifically, improving TCM body constitution may not necessarily improve sleep quality. This study aims to investigate potential variations in sleep quality among different TCM body constitution types in the Beijing high-speed railway crew.

## 2. Methods

This study was approved by the Scientific Research Ethics Committee of Beijing Shijitan Hospital, with the approval number sjtkyll-1x-2021(52) provided in Supplementary File S1 (Supplemental Digital Content, https://links.lww.com/MD/P959). Informed consent was obtained from all participants, with details documented in Supplementary File S2 (Supplemental Digital Content, https://links.lww.com/MD/P959).

### 2.1. Study design and participants

A cross-sectional study was conducted in the Beijing Shijitan Hospital, Beijing, China. Data was collected by using constitution in Chinese medicine questionnaire (CCMQ) and Pittsburgh sleep quality index (PSQI) from March 19, 2022 to November 20, 2023. In order to avoid selection bias, this study was conducted through random sampling from people who work in Beijing high-speed railway. Case inclusion criteria: Age ≥ 18 years; no organic pathology; no continuous medication use in the past month; Willing to participate and provide informed consent. Case exclusion criteria: Presence of psychiatric disorders or severe cognitive impairment; history of alcohol dependence; significant life events in the past 6 months; inability to cooperate with the researchers.

### 2.2. Sample size calculation

Based on the sample size calculation formula in survey methodology, *n* = sample size, *p* = prevalence (specifically noted as 38.2% in the China Sleep Quality Index Report^[15]^ for insomnia), *d* = margin of error (typically within ± 5%), *Z* = statistic (1.96 at 95% confidence level), so the minimum sample size is 363.


$$\begin{array}{l} \begin{array}{llll} & n={\left(Z_{\alpha /2}\right)}^{2}\times \frac{p(1-p)}{d^{2}} & n=1.96^{2}\times \frac{0.382\times (1-0.382)}{0.05^{2}} & n\approx 363 \end{array} \end{array}$$

By using 2 design effect 363 × 2 = 726 and adding 10% of the calculated sample size for non-response, therefore the final sample size was 799.

### 2.3. Data collection

The TCM body constitution assessment was conducted using the CCMQ, with details provided in Supplementary File S3 (Supplemental Digital Content, https://links.lww.com/MD/P959). This questionnaire was formulated by Professor Wang Qi from Beijing University of Chinese Medicine in 2005.^[16]^ Nowadays, it has been widely adopted as the standardized questionnaire for TCM body constitution assessment.^[17]^ TCM body constitution types include balanced constitution and imbalanced constitution. Balanced constitution was considered as a normal constitution, while the others were regarded as imbalanced constitution. The sleep quality evaluation was performed using the PSQI, with details provided in Supplementary File S4 (Supplemental Digital Content, https://links.lww.com/MD/P959). It was created in 1993 by sleep expert Buysse Dj from the University of Pittsburgh Medical Center. It was widely used to assess the quality of sleep.^[18]^

All participants completed the questionnaires under the guidance of researchers. Subsequently, 2 researchers independently entered the data into SPSS 26.0 and performed data quality control as follows: Logical verification: logical inconsistencies were checked, such as cases where participants reported “never experiencing insomnia” but also “daily use of sleeping pills”; consistency comparison: after double data entry, the 2 datasets were compared item by item, and discrepancies were flagged. The original questionnaires were then reviewed for verification and correction. Missing data handling: if any item had more than 10% missing values, the questionnaire was considered invalid and excluded (no questionnaires were excluded in this study). For items with 10% or fewer missing values, multiple imputation was used to fill in continuous variables, while categorical variables were retained as “missing” categories. Finally, after data checking and cleaning, the dataset was finalized and locked for subsequent statistical analysis.

### 2.4. Statistical analysis

All data were analyzed by SPSS Statistics 26.0. The Logit model, with the use of univariable and multivariable analyses, was used to identify relationship between TCM body constitution and sleep quality. All tests were two-tailed and the level of significance was set as *P* < .05. To ensure the robustness of the results, the Omnibus test of model coefficients was conducted to assess the goodness of fit (results of this study: *P* < .001), indicating that the regression model fit well and there was no evidence of significant deviation from the linear relationship. Furthermore, in order to visualize the results, the “basically yes” and “yes” were merged, “excellent sleep quality” and “good sleep quality” were merged, “fair sleep quality” and “BSQ” were merged.

## 3. Results

### 3.1. Sociodemographic characteristics of participants

**Figure 1.**
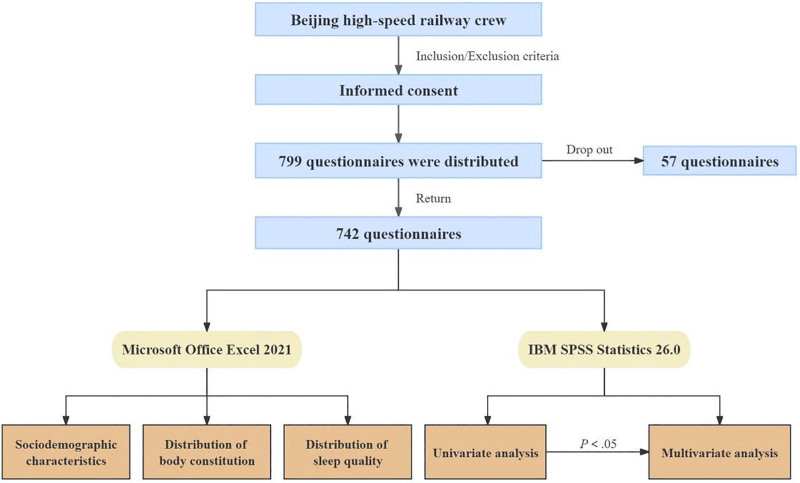
The schematic flow of this study.

799 questionnaires were distributed and 742 questionnaires were recovered (Fig. 1), with a valid questionnaire recovery rate of 92.87%. Sociodemographic characteristics of 742 cases of Beijing high-speed railway crew: sex: there were 75 males and 667 females, with a ratio of 1: 8.89; age: the minimum age was 20 years old, the maximum age was 34 years old and the median age was 25 years old; marriage: there were 512 participants unmarried and 230 participants married; academic degree: there were 32 participants had obtained secondary vocational school degree, 622 participants had obtained 3-year college degree and 88 participants had obtained 4-year college degree.

### 3.2. Distribution of TCM body constitution and sleep quality

**Figure 2.**
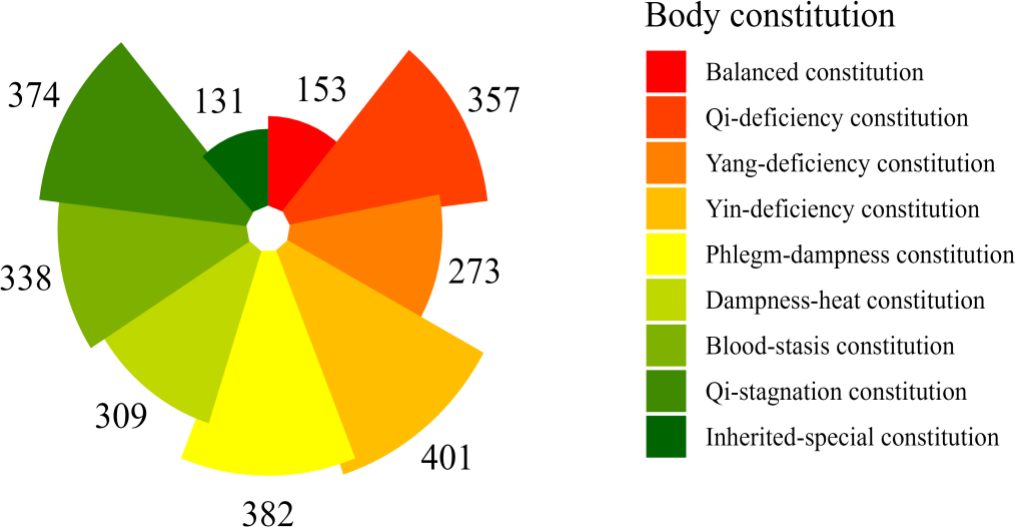
Distribution of traditional Chinese medicine body constitution.

Among the 742 cases of Beijing high-speed railway crew, there were 401 participants with Yin-deficiency constitution, 382 participants with Phlegm-dampness constitution, 374 participants with Qi-stagnation constitution, 357 participants with Qi-deficiency constitution, 338 participants with Blood-stasis constitution, 309 participants with Damp-heat constitution, 273 participants with Yang-deficiency constitution, 153 participants with Balanced constitution and 131 participants with Inherited-special constitution (Fig. 2).

**Figure 3.**
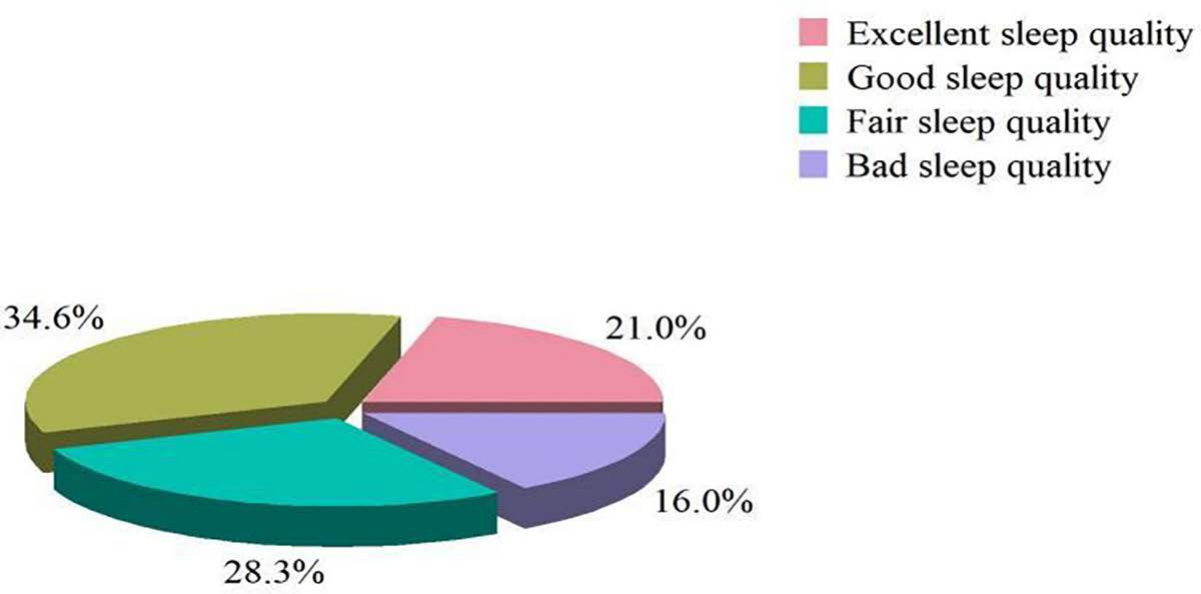
Distribution of sleep quality.

There were 156 participants with PSQI score of 0 to 5, which were judged as excellent sleep quality, accounting for 21.0% of the total population. There were 257 participants with PSQI score of 6 to 10, which were judged as good sleep quality, accounting for 34.6% of the total population. There were 210 participants with PSQI score of 11 to 15, which were judged as fair sleep quality, accounting for 28.3% of the total population. There were 119 participants with PSQI score of 16 to 21, which were judged as BSQ, accounting for 16.0% of the total population (Fig. 3).

### 3.3. Binary logistic regression analysis

The univariate analysis results showed that there was a correlation between sleep quality and Yang-deficiency constitution, Yin-deficiency constitution, phlegm-dampness constitution, dampness-heat constitution, blood-stasis constitution, Qi-stagnation constitution, inherited-special constitution, academic degree (*P* < .05). The multivariate analysis results showed that Yin-deficiency constitution (OR = 2.492, 95% CI = 1.824–3.405) and Qi-stagnation constitution (OR = 2.097, 95% CI = 1.429–3.076) were associated with the sleep quality (*P* < .001). These data are shown in Table 1.

**Table 1:** Binary logistic regression analysis to identify relationship between body constitution and sleep quality.

Variables	Univariate analysis		Multivariate analysis	
	OR (95% CI)	*P* value	OR (95% CI)	*P* value
Sex				
Male	1.000	–	–	–
Female	1.219 (0.749–1.982)	.425	–	–
Age	0.977 (0.930–1.026)	.348	–	–
Marriage				
Unmarried	1.000	–	–	–
Married	1.166 (0.853–1.593)	.336	–	–
Academic degree				
Secondary vocational school	1.000	–	–	–
3-yr college	0.466 (0.224–0.970)	**.041**	0.472 (0.219–1.017)	.055
4-yr college	0.435 (0.190–1.000)	.050	–	–
Balanced constitution				
No	1.000	–	–	–
Basically yes/yes	1.355 (0.949–1.935)	.095	–	–
Qi-deficiency constitution				
No	1.000	–	–	–
Basically yes/yes	1.237 (0.925–1.653)	.151	–	–
Yang-deficiency constitution				
No	1.000	–	–	–
Basically yes/yes	1.420 (1.051–1.917)	**.022**	1.179 (0.810–1.717)	.389
Yin-deficiency constitution				
No	1.000	–	–	–
Basically yes/yes	2.302 (1.708–3.102)	**<.001**	2.492 (1.824–3.405)	**<.001**
Phlegm-dampness constitution				
No	1.000	–	–	–
Basically yes/yes	1.505 (1.124–2.015)	**.006**	1.315 (0.962–1.798)	.086
Dampness-heat constitution				
No	1.000	–	–	–
Basically yes/yes	1.369 (1.020–1.837)	**.036**	0.890 (0.601–1.319)	.563
Blood-stasis constitution				
No	1.000	–	–	–
Basically yes/yes	1.459 (1.090–1.953)	**.011**	0.895 (0.587–1.363)	.605
Qi-stagnation constitution				
No	1.000	–	–	–
Basically yes/yes	1.990 (1.483–2.671)	**<.001**	2.097 (1.429–3.076)	**<.001**
Inherited-special constitution				
No	1.000	–	–	–
Basically yes/yes	1.619 (1.108–2.366)	**.013**	1.325 (0.858–2.047)	.204

*P* values <.05 are shown in bold.

## 4. Discussion

Previous studies have predominantly focused on the impact of occupational factors on sleep quality,^[19,20]^ with limited attention to individual differences in sleep quality due to constitutional types. For instance, shift work is known to disrupt circadian rhythms, leading to insomnia,^[21]^ but few studies have analyzed from a TCM perspective why some individuals are more susceptible to such disruptions. The novelty of this study lies in identifying that Yin-deficiency and Qi-stagnation constitutions are closely related to occupational sleep disorders among Beijing high-speed railway crew. This finding provides new insights into the occupational health management of high-speed railway crew.

Regarding working hours, studies have found that prolonged working hours significantly increase the risk of sleep disorders among British civil servants, particularly difficulties in falling asleep and shortened sleep duration.^[22]^ In terms of work stress, research has indicated that psychological stress and physical workload at work are important factors contributing to sleep disorders among healthcare professionals.^[23]^ Regarding shift patterns, studies have discovered that frequent switching between different shifts in a mixed shift schedule may lead to circadian rhythm disruption, thereby affecting the sleep quality of nurses.^[24]^ Additionally, research has shown that sleep disorders among firefighters are closely related to work stress, shift work, and psychological trauma.^[25]^ Therefore, working hours, work stress, and shift patterns are significant factors leading to occupational sleep disorders. High-speed railway crew, due to the unique nature of their work, are also susceptible to these factors and may experience sleep disorders as a result.

Yin-deficiency constitution is a type of TCM body constitution that is mainly characterized by a deficiency of body fluid.^[26]^ Due to the high requirements of work, high-speed railway crew usually need to think about more things. According to the theory of TCM, plenty of thinking depletes heart Yin,^[27]^ so it is easy to form a Yin-deficiency constitution in the long run. According to the theory of TCM, the mechanism of sleep disorder caused by Yin-deficiency constitution is mainly in the 2 aspects: Yin fluid has the functions of moisten, nourish and tranquilize. Due to the deficiency of Yin fluid, a person with a Yin-deficiency constitution is unable to adequately nourish the heart. The heart is associated with the spirit in TCM theory and the heart spirit refers to a person’s mental state and conscious activities. People with a Yin-deficiency constitution are often have sleep problems due to deficiency of heart Yin, which fails to nourish the heart to tranquilize^[28]^; Sleep disorder is often associated with Yin-deficiency or excessive Yang. Based on a mutually constrain relationship between Yin and Yang,^[29,30]^ people with a Yin-deficiency are often accompanied by a relatively high level of Yang. Excessive Yang Qi leads to internal heat in the body, resulting in symptoms such as dysphoria, tidal fever and night sweating, which also affect the quality of sleep.^[31]^

Qi-stagnation constitution is a type of TCM constitution that is mainly characterized by depression, anxiety, fragile,^[26]^ etc. In TCM theory, the formation of Qi-stagnation constitution is usually related to emotion and mental factors.^[32]^ High-speed railway crew need to face various kinds of passengers, which may cause them to bear so much stress. Finally, it often leads to depression and unstable emotions.^[33]^ In addition, long working hours,^[34]^ high standard of work requirements,^[35,36]^ frequent night shifts^[37]^ and irregular work and rest^[38,39]^ may cause high mental stress, leading to Qi movement stagnation, thus forming the Qi-stagnation constitution. According to the theory of TCM, the mechanism of sleep disorder caused by Qi-stagnation constitution is mainly in the 2 aspects: Qi can promotes the circulation of blood,^[40]^ so Qi movement stagnation will lead to blood stasis. People with Qi-stagnation constitution are prone to sleep disorder due to poor circulation of Qi and blood. The heart isn’t nourished by sufficient Qi and blood, resulting in restlessness; People with Qi-stagnation constitution are often accompanied by migraine,^[41]^ depression,^[42]^ and anxiety.^[43]^ These symptoms will also make people feel uncomfortable when they lying down to rest, thus affecting the sleep quality.

Our study has several limitations. First, the cross-sectional design is unable to establish a causal relationship between TCM body constitution and sleep quality. Second, the current calculation formula does not account for subgroup analysis, which may lead to insufficient samples in some categories for statistical analysis. Third, relying on self-reported questionnaires may introduce recall bias,^[44]^ thereby affecting the accuracy of the data. Fourth, due to limitations in data availability, other potential confounding factors, such as working hours and environmental conditions, could not be adjusted. Fifth, the results of this study were obtained from a sample of high-speed railway crew in Beijing, China, and thus may not be fully generalizable to other populations of high-speed railway crew in different countries. Future studies can adopt a longitudinal study design to investigate the causal relationship between TCM body constitution and sleep quality among high-speed railway crew while controlling for all known confounding variables. Additionally, future research could expand the sample size to include participants from different regions and countries to improve the generalizability of the findings. Nevertheless, the results of this study may still provide valuable insights for policymakers in the high-speed railway sector, alerting them to focus on the sleep quality of high-speed railway crew with Yin-deficiency constitution and Qi-stagnation constitution. This study also serves as a typical case for the application of TCM in occupational health surveillance.

## 5. Conclusions

This study reveals a significant association between Yin-deficiency and Qi-stagnation constitutions and poor sleep quality among Beijing high-speed railway crew. These findings highlight the potential utility of TCM body constitution assessment in identifying individuals at risk for sleep disorder. However, the cross-sectional design limits causal inference, and future longitudinal studies are needed to validate these associations and explore the effectiveness of TCM interventions in this occupational setting.

## Acknowledgments

We gratefully acknowledge the financial support provided by the National Administration of Traditional Chinese Medicine of China.

## Author contributions

**Conceptualization:** Bozhen Huang, Meijiao Zhou, Min Jiang.

**Formal analysis:** Meijiao Zhou, Shanshan Song, Lei Liu.

**Investigation:** Bozhen Huang, Meijiao Zhou, Shanshan Song, Guoyu Wang, Xinxin Liu.

**Methodology:** Bozhen Huang, Meijiao Zhou, Min Jiang.

**Project administration:** Bozhen Huang, Shanshan Song, Min Jiang.

**Visualization:** Lei Liu, Liang Wang, Xinqian Liu, Jiaxuan Lyu.

**Writing – original draft:** Bozhen Huang, Meijiao Zhou, Liang Wang, Fangzhou Jin.

**Writing – review & editing:** Bozhen Huang, Meijiao Zhou, Liang Wang, Xinqian Liu.

## Supplementary Material


