# Supplementary material for: Relationship between traditional Chinese medicine body constitution and sleep quality among high-speed railway crew in Beijing, China: A cross-sectional study
Source: Medicine (Baltimore). 2025 Sep 12;104(37):e44563. doi: 10.1097/MD.0000000000044563 (PMC12440421; doi:10.1097/MD.0000000000044563)
Supplement: Supplementary file 1 [file medi-104-e44563-s001.pdf]

首都医科大学附属北京世纪坛医院科学研究伦理委员会

科学研究课题审批件

sjtkyll-lx-2021(52)

<table>
<tr><td rowspan="6">评审项目</td><td>项目名称</td><td colspan="3">高铁乘务员中医体质和睡眠状态的相关性研究</td></tr>
<tr><td>课题来源</td><td colspan="3">☐国际合作课题 ☐国家级科研课题 ☑省部级科研课题
☐校局级科研课题 ☐医院科研课题 ☐其它:</td></tr>
<tr><td>课题负责人</td><td colspan="3">吴欣</td></tr>
<tr><td>职称</td><td>副主任医师</td><td>课题编号</td><td>J2020Z606</td></tr>
<tr><td>科室</td><td>中医科</td><td>起止时间</td><td>202104-202204</td></tr>
<tr><td>联系电话</td><td>63926519</td><td>参加研究人员</td><td>无</td></tr>
<tr><td>受理审查文件</td><td colspan="4">☑ 1. 课题任务合同书（封面及有印章部分的复印件，基金项目）
☐ 2. 专家推荐书 3 份（研究者自发项目）
☐ 3. 项目主持单位伦理批件复印件（如本单位为合作单位）
☐ 4. 研究合同（横向课题）
☑ 5. 科学研究方案（20210413 第一版）
☑ 6. 病例报告表（20210413 第一版）
☑ 7. 知情同意书（20210413 第一版）
☑ 8. 招募广告（20210413 第一版）
☐ 9. 免知情同意申请书（请在此处注明本方案的版本号和版本日期）
☐ 10. 其他:</td></tr>
<tr><td rowspan="2">委员意见</td><td colspan="4">出席 10 人 ｜ 投票 10 人 ｜ 回避 0 人 ｜ 批准 10 人 ｜ 不批准 0 人</td></tr>
<tr><td colspan="4">修正后批准 0 人 ｜ 修正后再审 0 人 ｜ 暂停或终止研究 0 人</td></tr>
<tr><td>初审结论</td><td colspan="4">☑批准 ☐不批准 ☐终止或暂停已批准的试验
☐作必要的修正后批准，详见审查意见函 ☐作必要的修正后再审，详见审查意见函</td></tr>
<tr><td>初审日期</td><td colspan="4">2021.6.3</td></tr>
<tr><td>复审提交材料</td><td colspan="4">无</td></tr>
<tr><td>复审日期</td><td>无</td><td>复审委员 无</td><td colspan="2">复审意见 无</td></tr>
<tr><td>审批意见</td><td colspan="4">批准该课题项目开展。请遵循 GCP 原则、遵循伦理委员会批准的方案开展临床研究，保护受试者的健康与权益。

主任委员/授权者签名:
科学研究伦理委员会（盖章）:
日期:2021.6.10</td></tr>
<tr><td>备注</td><td colspan="4">该研究进行过程中将接受伦理委员会的持续审查? ☑ 是☐否
审查频度为研究批准之日起☐3 个月 ☐6 个月 ☑12 个月
本批件有效期为 2021 年 6 月 10 日-2022 年 6 月 9 日，如此项目在批件有效期内未能启动临床研究，本批件作废，需要重新提交伦理审查申请。</td></tr>
</table>

知情同意书

尊敬的受访者:

您好！现邀请您参加由北京世纪坛医院举办的“高铁乘务员中医体质与睡眠质量相关性研究”。在同意参加之前，如果您能够仔细阅读以确保您充分了解研究的性质、目的和过程，我们将会感到欣慰。

1.研究目的:

本研究旨在探讨中医体质在预测北京高铁乘务员睡眠障碍中的作用，以改善其睡眠质量，提高其工作效率，助力高铁行业的发展。

2.程序和时间:

您将被要求回答一系列问题，预计花费您大约 15 分钟。请您在填写问卷时认真思考，并根据您的实际情况回答问题。

3.隐私保护:

您的个人信息将被严格保密，且在报告研究结果时将进行匿名处理。仅研究者能够访问您的答卷，并不会透露您的身份。

4.自愿参与:

参与本研究是完全自愿的，您有权随时退出，而不会受到任何不利影响。如果您决定退出，您的数据将不被使用，并将从研究中删除。

同意声明

我已经阅读了有关本研究的介绍，而且有机会与研究者讨论并提出问题。我提出的所有问题都得到了满意的答复，最后，我明确表示同意参与本次问卷调查。

患者签名：__________________ 患者电话：__________________ 日期：______________

我确认已向患者解释本研究的详细情况，并给其一份签署过的知情同意书副本。

医者签名：__________________ 医者电话：__________________ 日期：______________

联系电话：010-63926603

通讯地址：北京市海淀区羊坊店铁医路10号

中医体质分类与判定自测表
(中华中医药学会标准)

计分方法与评判标准：

每条题目下设5级答案，由无到有的倾向性给出1～5分的分值(其中标有*的条目为逆向计分项目)，以单选方式选择，然后对每类的原始分采用简单求和法。

原始分数＝各条题目分支的累加和。

转化分数＝[(原始分－条目数)/(条目数×4)]×100。

平和体质与偏颇体质判定标准为：

体质类型	条件	判定结果
平和体质	转化分≥60分	是
	其他8种体质转化分均<30分	
	转化分≥60分	基本是
	其他8种体质转化分均<40分	
	不满足上述条件者	否
偏颇体质	转化分≥40分	是
	转化分30～39分	倾向是
	转化分<30分	否

阳虚体质

请根据近一年的体验和感觉，回答以下问题	没有(根本不)	很少(有一点)	有时(有些)	经常(相当)	总是(非常)
(1)您手脚发凉吗？	1	2	3	4	5
(2)您胃脘部、背部或腰膝部怕冷吗？	1	2	3	4	5
(3)您感到怕冷、衣服比别人穿得多吗？	1	2	3	4	5
(4)您比一般人耐受不了寒冷(冬天的寒冷，夏天的冷空调、电扇等)吗？	1	2	3	4	5
(5)您比别人容易患感冒吗？	1	2	3	4	5
(6)您吃(喝)凉的东西会感到不舒服或者怕吃(喝)凉东西吗？	1	2	3	4	5
(7)您受凉或吃(喝)凉的东西后，容易腹泻(拉肚子)吗？	1	2	3	4	5
判断结果：□是　□倾向是　□否					

阴虚体质

请根据近一年的体验和感觉，回答以下问题	没有<br>（根本不）	很少<br>（有一点）	有时<br>（有些）	经常<br>（相当）	总是<br>（非常）
(1)您感到手脚心发热吗？	1	2	3	4	5
(2)您感觉身体、脸上发热吗？	1	2	3	4	5
(3)您皮肤或口唇干吗？	1	2	3	4	5
(4)您口唇的颜色比一般人红吗？	1	2	3	4	5
(5)您容易便秘或大便干燥吗？	1	2	3	4	5
(6)您面部两颧潮红或偏红吗？	1	2	3	4	5
(7)您感到眼睛干涩吗？	1	2	3	4	5
(8)您感到口干咽燥、总想喝水吗？	1	2	3	4	5
判断结果：□是 □倾向是 □否					

气虚体质

请根据近一年的体验和感觉，回答以下问题	没有<br>（根本不）	很少<br>（有一点）	有时<br>（有些）	经常<br>（相当）	总是<br>（非常）
(1)您容易疲乏吗？	1	2	3	4	5
(2)您容易气短(呼吸短促，接不上气)吗？	1	2	3	4	5
(3)您容易心慌吗？	1	2	3	4	5
(4)您容易头晕或站起时晕眩吗？	1	2	3	4	5
(5)您比别人容易患感冒吗？	1	2	3	4	5
(6)您喜欢安静、懒得说话吗？	1	2	3	4	5
(7)您说话声音低弱无力吗？	1	2	3	4	5
(8)您活动量稍大就容易出虚汗吗？	1	2	3	4	5
判断结果：□是 □倾向是 □否					

痰湿体质

请根据近一年的体验和感觉，回答以下问题	没有<br>（根本不）	很少<br>（有一点）	有时<br>（有些）	经常<br>（相当）	总是<br>（非常）
(1)您感到胸闷或腹部胀满吗？	1	2	3	4	5
(2)您感到身体沉重不轻松或不爽快吗？	1	2	3	4	5
(3)您腹部肥满松软吗？	1	2	3	4	5
(4)您有额部油脂分泌多的现象吗？	1	2	3	4	5
(5)您上眼睑比别人肿(上眼睑有轻微隆起的现象)吗？	1	2	3	4	5
(6)您嘴里有黏黏的感觉吗？	1	2	3	4	5

续表

请根据近一年的体验和感觉，回答以下问题	没有（根本不）	很少（有一点）	有时（有些）	经常（相当）	总是（非常）
(7)您平时痰多，特别是咽喉部总感到有痰堵着吗？	1	2	3	4	5
(8)您舌苔厚腻或有舌苔厚厚的感觉吗？	1	2	3	4	5
判断结果：□是 □倾向是 □否					

湿热体质

请根据近一年的体验和感觉，回答以下问题	没有（根本不）	很少（有一点）	有时（有些）	经常（相当）	总是（非常）
(1)您面部或鼻部有油腻感或者油亮发光吗？	1	2	3	4	5
(2)您容易生痤疮或疮疖吗？	1	2	3	4	5
(3)您感到口苦或嘴里有异味吗？	1	2	3	4	5
(4)您大便黏滞不爽、有解不尽的感觉吗？	1	2	3	4	5
(5)您小便时尿道有发热感、尿色浓（深）吗？	1	2	3	4	5
(6)您带下色黄（白带颜色发黄）吗？（限女性回答）	1	2	3	4	5
(7)您的阴囊部位潮湿吗？（限男性回答）	1	2	3	4	5
判断结果：□是 □倾向是 □否					

瘀血体质

请根据近一年的体验和感觉，回答以下问题	没有（根本不）	很少（有一点）	有时（有些）	经常（相当）	总是（非常）
(1)您的皮肤在不知不觉中会出现青紫瘀斑（皮下出血）吗？	1	2	3	4	5
(2)您两颧部有细微红丝吗？	1	2	3	4	5
(3)您身体上有哪里疼痛吗？	1	2	3	4	5
(4)您面色晦暗或容易出现褐斑吗？	1	2	3	4	5
(5)您容易有黑眼圈吗？	1	2	3	4	5
(6)您容易忘事（健忘）吗？	1	2	3	4	5
(7)您口唇颜色偏暗吗？	1	2	3	4	5
判断结果：□是 □倾向是 □否					

气郁体质

请根据近一年的体验和感觉，回答以下问题	没有<br>（根本不）	很少<br>（有一点）	有时<br>（有些）	经常<br>（相当）	总是<br>（非常）
(1)您感到闷闷不乐、情绪低沉吗？	1	2	3	4	5
(2)您容易精神紧张、焦虑不安吗？	1	2	3	4	5
(3)您多愁善感、感情脆弱吗？	1	2	3	4	5
(4)您容易感到害怕或受到惊吓吗？	1	2	3	4	5
(5)您胁肋部或乳房胀痛吗？	1	2	3	4	5
(6)您无缘无故叹气吗？	1	2	3	4	5
(7)您咽喉部有异物感，且吐之不出、咽之不下吗？	1	2	3	4	5
判断结果：□是　　□倾向是　　□否					

特禀体质

请根据近一年的体验和感觉，回答以下问题	没有<br>（根本不）	很少<br>（有一点）	有时<br>（有些）	经常<br>（相当）	总是<br>（非常）
(1)您没有感冒时也会打喷嚏吗？	1	2	3	4	5
(2)您没有感冒时也会鼻塞、流鼻涕吗？	1	2	3	4	5
(3)您有因季节变化、温度变化或异味等原因而咳喘的现象吗？	1	2	3	4	5
(4)您容易过敏（对药物、食物、气味、花粉或在季节交替、气候变化时）吗？	1	2	3	4	5
(5)您的皮肤容易起荨麻疹（风团、风疹块、风疙瘩）吗？	1	2	3	4	5
(6)您的皮肤因过敏出现过紫癜（紫红色瘀点、瘀斑）吗？	1	2	3	4	5
(7)您的皮肤一抓就红，并出现抓痕吗？	1	2	3	4	5
判断结果：□是　　□倾向是　　□否					

平和体质

请根据近一年的体验和感觉，回答以下问题	没有<br>（根本不）	很少<br>（有一点）	有时<br>（有些）	经常<br>（相当）	总是<br>（非常）
(1)您精力充沛吗？	1	2	3	4	5
(2)您容易疲乏吗？ *	5	4	3	2	1
(3)您说话声音低弱无力吗？ *	5	4	3	2	1
(4)您感到闷闷不乐、情绪低沉吗？ *	5	4	3	2	1

续表

请根据近一年的体验和感觉，回答以下问题	没有（根本不）	很少（有一点）	有时（有些）	经常（相当）	总是（非常）
(5)您比一般人耐受不了寒冷(冬天的寒冷，夏天的冷空调、电扇等)吗？ *	5	4	3	2	1
(6)您能适应外界自然和社会环境的变化吗？	1	2	3	4	5
(7)您容易失眠吗？ *	5	4	3	2	1
(8)您容易忘事(健忘)吗？ *	5	4	3	2	1
判断结果：□是　□倾向是　□否					

示例：

示例1：某人各体质类型转化分如下：平和体质75分，气虚体质56分，阳虚体质27分，阴虚体质25分，痰湿体质12分，湿热体质15分，血瘀体质20分，气郁体质18分，特禀体质10分。根据判定标准，虽然平和体质转化分≥60分，但其他8种体质转化分并未全部<40分，其中气虚转化分≥40分，故此人不能判定为平和体质，应判定为气虚体质。

示例2：某人各体质类型转化分如下：平和体质75分，气虚体质16分，阳虚体质25分，痰湿体质32分，湿热体质15分，血瘀体质20分，气郁体质18分，特禀体质10分。根据判定标准，虽然平和体质转化分≥60分，且其他8种体质转化分均<40分，可判定为基本是平和体质，同时，痰湿体质转化分在30～39分之间，可判定为痰湿体质倾向，故此人最终体质判定结果基本是平和体质，有痰湿体质倾向。

问题：

1.您的体质类型是？

2.您的体质类型倾向是？

3.您的体质类型容易出现什么症状，应当注意哪些方面的调护？

Appendix. Pittsburgh Sleep Quality Index (PSQI)

Name ______________________ ID # __________ Date __________ Age ______

Instructions:

The following questions relate to your usual sleep habits during the past month *only*. Your answers should indicate the most accurate reply for the *majority* of days and nights in the past month. Please answer all questions.

1. During the past month, when have you usually gone to bed at night?

   USUAL BED TIME __________

2. During the past month, how long (in minutes) has it usually take you to fall asleep each night?

   NUMBER OF MINUTES __________

3. During the past month, when have you usually gotten up in the morning?

   USUAL GETTING UP TIME __________

4. During the past month, how many hours of *actual sleep* did you get at night? (This may be different than the number of hours you spend in bed.)

   HOURS OF SLEEP PER NIGHT __________

For each of the remaining questions, check the one best response. Please answer *all* questions.

5. During the past month, how often have you had trouble sleeping because you...

   (a) Cannot get to sleep within 30 minutes
   Not during the past month ____ Less than once a week ____ Once or twice a week ____ Three or more times a week ____

   (b) Wake up in the middle of the night or early morning
   Not during the past month ____ Less than once a week ____ Once or twice a week ____ Three or more times a week ____

   (c) Have to get up to use the bathroom
   Not during the past month ____ Less than once a week ____ Once or twice a week ____ Three or more times a week ____

   (d) Cannot breathe comfortably
   Not during the past month ____ Less than once a week ____ Once or twice a week ____ Three or more times a week ____

   (e) Cough or snore loudly
   Not during the past month ____ Less than once a week ____ Once or twice a week ____ Three or more times a week ____

   (f) Feel too cold
   Not during the past month ____ Less than once a week ____ Once or twice a week ____ Three or more times a week ____

   (g) Feel too hot
   Not during the past month ____ Less than once a week ____ Once or twice a week ____ Three or more times a week ____

   (h) Had bad dreams
   Not during the past month ____ Less than once a week ____ Once or twice a week ____ Three or more times a week ____

   (i) Have pain
   Not during the past month ____ Less than once a week ____ Once or twice a week ____ Three or more times a week ____

(j) Other reason(s), please describe ____

____

How often during the past month have you had trouble sleeping because of this?

Not during the past month _____ Less than once a week _____ Once or twice a week _____ Three or more times a week _____

6. During the past month, how would you rate your sleep quality overall?

Very good _______
Fairly good _______
Fairly bad _______
Very bad _______

7. During the past month, how often have you taken medicine (prescribed or "over the counter") to help you sleep?

Not during the past month _____ Less than once a week _____ Once or twice a week _____ Three or more times a week _____

8. During the past month, how often have you had trouble staying awake while driving, eating meals, or engaging in social activity?

Not during the past month _____ Less than once a week _____ Once or twice a week _____ Three or more times a week _____

9. During the past month, how much of a problem has it been for you to keep up enough enthusiasm to get things done?

No problem at all _______
Only a very slight problem _______
Somewhat of a problem _______
A very big problem _______

10. Do you have a bed partner or roommate?

No bed partner or roommate _______
Partner/roommate in other room _______
Partner in same room, but not same bed _______
Partner in same bed _______

If you have a roommate or bed partner, ask him/her how often in the past month you have had...

(a) Loud snoring

Not during the past month _____ Less than once a week _____ Once or twice a week _____ Three or more times a week _____

(b) Long pauses between breaths while asleep

Not during the past month _____ Less than once a week _____ Once or twice a week _____ Three or more times a week _____

(c) Legs twitching or jerking while you sleep

Not during the past month _____ Less than once a week _____ Once or twice a week _____ Three or more times a week _____

(d) Episodes of disorientation or confusion during sleep

Not during the past month _____ Less than once a week _____ Once or twice a week _____ Three or more times a week _____

(e) Other restlessness while you sleep; please describe ______________________________

____

Not during the past month _____ Less than once a week _____ Once or twice a week _____ Three or more times a week _____

Scoring Instructions for the Pittsburgh Sleep Quality Index

The Pittsburgh Sleep Quality Index (PSQI) contains 19 self-rated questions and 5 questions rated by the bed partner or roommate (if one is available). Only self-rated questions are included in the scoring. The 19 self-rated items are combined to form seven "component" scores, each of which has a range of 0-3 points. In all cases, a score of "0" indicates no difficulty, while a score of "3" indicates severe difficulty. The seven component scores are then added to yield one "global" score, with a range of 0-21 points, "0" indicating no difficulty and "21" indicating severe difficulties in all areas.

Scoring proceeds as follows:

Component 1: Subjective sleep quality

Examine question #6, and assign scores as follows:

Response	Component 1 score
"Very good"	0
"Fairly good"	1
"Fairly bad"	2
"Very bad"	3

Component 1 score: ______

Component 2: Sleep latency

1. Examine question #2, and assign scores as follows:

Response	Score
≤ 15 minutes	0
16-30 minutes	1
31-60 minutes	2
> 60 minutes	3

Question #2 score: ______

2. Examine question #5a, and assign scores as follows:

Response	Score
Not during the past month	0
Less than once a week	1
Once or twice a week	2
Three or more times a week	3

Question #5a score: ______

3. Add #2 score and #5a score

Sum of #2 and #5a: ______

4. Assign component 2 score as follows:

Sum of #2 and #5a	Component 2 score
0	0
1-2	1
3-4	2
5-6	3

Component 2 score: ______

Component 3: Sleep duration

Examine question #4, and assign scores as follows:

Response	Component 3 score
> 7 hours	0
6-7 hours	1
5-6 hours	2
< 5 hours	3

Component 3 score: ______

Component 4: Habitual sleep efficiency

(1) Write the number of hours slept (question # 4) here: ________

(2) Calculate the number of hours spent in bed:

Getting up time (question # 3): ________

− Bedtime (question # 1): ________

Number of hours spent in bed: ________

(3) Calculate habitual sleep efficiency as follows:

(Number of hours slept/Number of hours spent in bed) × 100 = Habitual sleep efficiency (%)

(______/______) × 100 = ________%

(4) Assign component 4 score as follows:

Habitual sleep efficiency %	Component 4 score
> 85%	0
75-84%	1
65-74%	2
< 65%	3

Component 4 score: ________

Component 5: Sleep disturbances

(1) Examine questions # 5b-5j, and assign scores for *each* question as follows:

Response	Score
Not during the past month	0
Less than once a week	1
Once or twice a week	2
Three or more times a week	3

#5b score ________
c score ________
d score ________
e score ________
f score ________
g score ________
h score ________
i score ________
j score ________

(2) Add the scores for questions # 5b-5j:

Sum of # 5b-5j: ________

(3) Assign component 5 score as follows:

Sum of # 5b-5j	Component 5 score
0	0
1-9	1
10-18	2
19-27	3

Component 5 score: ________

Component 6: Use of sleeping medication

Examine question # 7 and assign scores as follows:

Response	Component 6 score
Not during the past month	0
Less than once a week	1
Once or twice a week	2
Three or more times a week	3

Component 6 score: ________

Component 7: Daytime dysfunction

(1) Examine question # 8, and assign scores as follows:

Response	Score
Never	0
Once or twice	1
Once or twice each week	2
Three or more times each week	3

Question # 8 score: ______

(2) Examine question # 9, and assign scores as follows:

Response	Score
No problem at all	0
Only a very slight problem	1
Somewhat of a problem	2
A very big problem	3

Question # 9 score: ______

(3) Add the scores for question # 8 and # 9:

Sum of #8 and #9: ______

(4) Assign component 7 score as follows:

Sum of # 8 and #9	Component 7 score
0	0
1-2	1
3-4	2
5-6	3

Component 7 score: ______

Global PSQI Score

Add the seven component scores together:

Global PSQI Score: ______
